# Regulation of plant glycolysis and the tricarboxylic acid cycle by posttranslational modifications

**DOI:** 10.1111/tpj.70142

**Published:** 2025-04-04

**Authors:** Ke Zheng, Maria del Pilar Martinez, Maroua Bouzid, Manuel Balparda, Markus Schwarzländer, Veronica G. Maurino

**Affiliations:** ^1^ Plant Energy Biology Lab Institute of Plant Biology and Biotechnology (IBBP), University of Münster Schlossplatz 8 Münster 48145 Germany; ^2^ Molecular Plant Physiology Institute of Cellular Molecular Botany (IZMB), University of Bonn Kirschallee 1 Bonn 53115 Germany

**Keywords:** glycolysis, tricarboxylic acid cycle, posttranslational modifications, central carbon metabolism, functional changes

## Abstract

Plant glycolysis and the tricarboxylic acid (TCA) cycle are key pathways of central carbon metabolism. They facilitate energy transformation, provide redox balance, and supply the building blocks for biosynthetic processes that underpin plant survival, growth, and productivity. Yet, rather than acting as static pathways, the fluxes that are mediated by the enzymes involved form a branched network. Flux modes can change flexibly to match cellular demands and environmental fluctuations. Several of the enzymes involved in glycolysis and the TCA cycle have been identified as targets of posttranslational modifications (PTMs). PTMs can act as regulators to facilitate changes in flux by rapidly and reversibly altering enzyme organization and function. Consequently, PTMs enable plants to rapidly adjust their metabolic flux landscape, match energy and precursor provision with the changeable needs, and enhance overall metabolic flexibility. Here, we review the impact of different PTMs on glycolytic and TCA cycle enzymes, focusing on modifications that induce functional changes rather than the mere occurrence of PTMs at specific sites. By synthesizing recent findings, we provide a foundation for a system‐level understanding of how PTMs choreograph the remarkable flexibility of plant central carbon metabolism.

## Significance Statement

This review explores how posttranslational modifications (PTMs) influence enzymes in glycolysis and the tricarboxylic acid cycle, key pathways that support plant growth and productivity. By examining the functional impact of PTMs on metabolic flux regulation, this work contributes to a deeper understanding of how plants maintain metabolic flexibility in response to fluctuating internal and external conditions, with implications for improving plant resilience and metabolic optimization.

## INTRODUCTION

**Figure 1:**
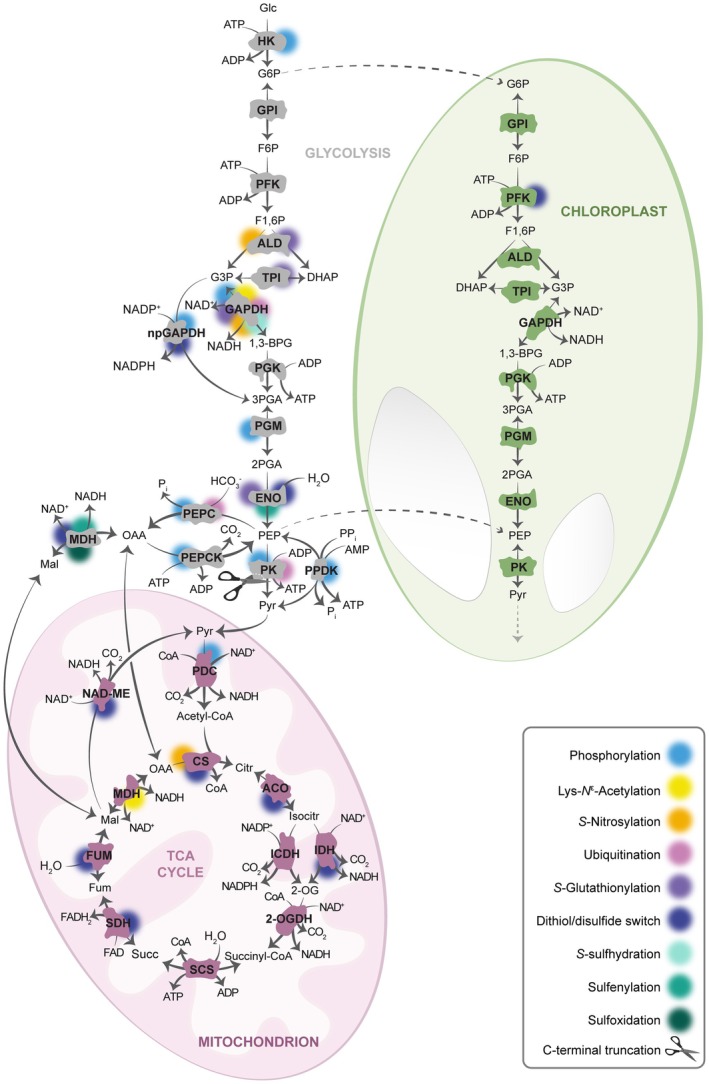
PTMs of proteins of plant glycolysis and the TCA cycle. 1,3‐BPG, 1,3‐bisphosphoglycerate; 2‐OG, 2‐oxoglutarate; 2‐OGDH, 2‐oxoglutarate dehydrogenase; 2PGA, 2‐phosphoglycerate; 3PGA, 3‐phosphoglycerate; ACO, aconitase; ADP, adenosine diphosphate; ALD, aldolase; AMP, adenosine monophosphate; ATP, adenosine triphosphate; Citr, citrate; CoA, coenzyme A; CS, citrate synthase; DHAP, dihydroxyacetone phosphate; ENO, enolase; F1,6P, fructose‐1,6‐bisphosphate; F6P, fructose‐6‐phosphate; FAD, flavin adenine dinucleotide; FUM, fumarase; Fum, fumarate; G3P, glyceraldehyde‐3‐phosphate; G6P, glucose‐6‐phosphate; GAPDH, glyceraldehyde‐3‐phosphate dehydrogenase; Glc, glucose; GPI, glucose‐6‐phosphate isomerase; HK, hexokinase; IDH, NAD^+^‐isocitrate dehydrogenase; Isocitr, isocitrate; Mal, malate; MDH, malate dehydrogenase; NAD‐ME, NAD^+^‐malic enzyme; NADP, nicotinamide adenine dinucleotide phosphate; npGAPDH, non‐phosphorylating glyceraldehyde‐3‐phosphate dehydrogenase; OAA, oxaloacetate; PDC, pyruvate dehydrogenase complex; PEP, phosphoenolpyruvate; PEPC, phosphoenolpyruvate carboxylase; PEPCK, phosphoenolpyruvate carboxykinase; PFK, phosphofructokinase; PGK, phosphoglycerate kinase; PGM, phosphoglycerate mutase; P_i_, inorganic phosphate; PK, pyruvate kinase; PPDK, pyruvate, orthophosphate dikinase; PP_i_, inorganic pyrophosphate; PTM, posttranslational modification; Pyr, pyruvate; SCS, succinyl‐CoA synthetase; SDH, succinate dehydrogenase; Succ, succinate; TCA, tricarboxylic acid; TPI, triose phosphate isomerase.

Plant central carbon metabolism is underpinned by glycolysis and the tricarboxylic acid (TCA) cycle (Figure 1). These interconnected metabolic routes serve at least two major roles: first, to fuel the mitochondrial electron transport chain to synthesize adenosine triphosphate (ATP) by providing the required reducing power; and second, to supply the cell with the fundamental molecular building blocks required for plant growth, development, survival, and reproduction. Glycolysis, as the initial reaction sequence of glucose catabolism, converts glucose into pyruvate, generating ATP and reducing equivalents, most importantly nicotinamide adenine dinucleotide (NADH) (Figure 1). This metabolic process takes place in the cytosol and the plastid stroma of green and non‐green plant cells in which the requirements of energy and precursors may differ markedly. In both compartments, glycolysis contains a complete set of the required enzymes each and can exchange key intermediates through the action of a set of specific transporters in the inner plastid envelope (Heldt & Piechulla, 2011). The primary function of glycolysis in chloroplasts and plastids from heterotrophic tissues is the degradation of starch to provide energy and primary metabolites essential for anabolic processes, such as fatty acid‐ and amino acid biosynthesis (Andre et al., 2007; Andriotis et al., 2010; Baud et al., 2007). Pyruvate produced by cytosolic glycolysis can be imported into the plastid stroma by BILE ACID:SODIUM SYMPORTER FAMILY PROTEIN 2 (BASS2) where it – together with the pyruvate produced by stromal glycolysis and Rubisco (Evans et al., 2024) – is a substrate for the synthesis of fatty acids, terpenoids, and branched‐chain amino acids (Furumoto et al., 2011). Cytosolic pyruvate can also be transported into the mitochondrial matrix by the mitochondrial pyruvate carrier (MPC) (Le et al., 2021). After oxidative decarboxylation linked to NAD reduction and synthesis of acetyl‐CoA, two of the carbon atoms can enter the TCA cycle to release reducing equivalents and energy in the form of NADH and ATP (Figure 1). Depending on the state of the metabolic network, the reducing equivalents may be exported via metabolic shuttle systems or utilized by the electron carriers of the mitochondria electron transport chain (mETC), which in turn facilitate ATP synthesis through oxidative phosphorylation and dissipate a proportion of the energy as heat (Sweetlove et al., 2010; Taiz & Zeiger, 2010).

To maintain resource use efficiency and – probably even more importantly for plants – redox balance, while acclimating to changing environmental conditions, the tight regulation of these metabolic pathways is critical. Among these regulatory mechanisms, posttranslational modifications (PTMs) are key players in the modulation of enzyme activities and metabolic fluxes. PTMs include a chemically diverse set of covalent modifications that involve the addition, removal, or conversion of chemical groups by, for instance, phosphorylation, acetylation, methylation, ubiquitination, cysteine oxidation, and many others (Balparda et al., 2023). Compared to other regulatory mechanisms, PTMs offer a rapid, precise, and flexible means of modulating protein organization and function, enabling plants to rapidly adjust their central carbon metabolism to match metabolic demands under changing environmental conditions.

A rapidly growing body of research and several excellent reviews have shed light on the occurrence of PTMs in central carbon metabolism in plants (da Fonseca‐Pereira et al., 2021; Martí et al., 2020; Nunes‐Nesi et al., 2013; O'Leary & Plaxton, 2020; Yoshida et al., 2013). However, previous literature has mainly focused on specific steps of the metabolic pathways or individual PTM types, resulting in a fragmented perspective (Dixon et al., 2005; Ito et al., 2009; Lindermayr et al., 2005; Tovar‐Méndez et al., 2003). This review seeks to provide an understanding of the functional role of prominent PTMs of glycolytic and TCA cycle enzymes, to explore the dynamic interplay between PTMs and enzyme functionality in plant central carbon metabolism. It complements a recent review that has focused on the regulation of PTM‐mediated regulation of carbon assimilation pathways (Balparda et al., 2023).

## REGULATION OF GLYCOLYSIS AND ITS ASSOCIATED ENZYMES BY PTMs

Glycolysis converts hexose sugars, such as glucose, into pyruvate (Figure 1). This process plays a major role in supplying the cell with usable energy and can serve as the primary source of ATP under certain conditions, such as hypoxia (Cho et al., 2021) or in specific tissues such as stem cells (Mira et al., 2023; Weits et al., 2019). Under standard respiratory conditions, glycolysis‐derived pyruvate fuels the mitochondrial TCA cycle and oxidative phosphorylation. However, the significance of glycolysis extends beyond energy provision; its intermediates serve as essential building blocks for anabolic pathways, including amino acid biosynthesis and secondary metabolism.

In plants, glycolysis takes place in both the cytosol and plastids of heterotrophic and autotrophic cells (Figure 1), where the energy and precursor requirements differ. Distinct enzymes or enzyme isoforms are involved in these distinct pathways, each with unique regulatory properties tailored to the specific needs of different tissues and cellular contexts. The coordinated regulation between plastidial and cytosolic glycolysis is essential for shaping plant development, particularly in response to environmental changes. While phosphorylation and redox modifications of cysteine thiols are likely to be the predominant PTMs regulating plant glycolytic enzyme activities, emerging evidence suggests roles for other PTMs such as acetylation and ubiquitination. In this section, we provide an enzyme‐by‐enzyme summary of what is currently known about the regulation of glycolytic enzymes by the diverse array of PTMs that modulate their structure, activity, and interactions. We dedicate individual sections to highlighting enzymes for which regulation by PTMs has been established (Figure 1, Table 1). For most others, there is also evidence for the occurrence of PTMs, yet there is either no or not yet conclusive evidence for the functional implication of those modifications.

### Hexokinase

Hexokinase (HXK; EC 2.7.1.1) catalyzes the phosphorylation of glucose to form glucose‐6‐phosphate (G6P), which is the first step in glycolysis (Figure 1). Beyond its role in metabolism, hexokinase acts as a sensor for glucose levels. It helps regulate gene expression related to growth and development in response to sugar availability (Xiao et al., 2000).

Arabidopsis HXK1 undergoes *phosphorylation* at serine residues Ser184 and Ser186 (Mergner et al., 2020). These phosphorylation sites are proximal to serine Ser177, which is hypothesized to play a pivotal role in phosphoryl transfer during catalysis based on homology studies. When Ser177 is substituted with alanine in Arabidopsis, it yields a catalytically inactive form of HXK1, though this variant maintains its glucose‐sensing capability (Moore et al., 2003). Ser177 in Arabidopsis HXK1 is analogous to Ser158 in yeast HXK2 (Feng et al., 2015). This conserved serine residue is essential for catalytic activity by facilitating phosphoryl transfer during glucose phosphorylation (Arora et al., 1991; Feng et al., 2015; Heidrich et al., 1997).

### Phosphofructokinase

Phosphofructokinase (PFK) catalyzes the phosphorylation of fructose 6‐phosphate to fructose 1,6‐bisphosphate (F1,6P) (Figure 1) (Yoshida & Hisabori, 2021). Two distinct types of PFK exist: pyrophosphate‐dependent PFK (PP_i_‐PFK; EC 2.7.1.90) and ATP‐dependent PFK (ATP‐PFK; EC 2.7.1.11). Notably, only PP_i_‐PFK mediates a reversible reaction. Both forms of PFK work in parallel and predominantly reside in the cytosol, with some reports suggesting the localization of ATP‐PFK in plastids of various plant species (Turner & Plaxton, 2003; Winkler et al., 2007). Arabidopsis contains seven genes encoding different PFK isoforms (PFK1‐7), with isoforms 4 and 5 possessing transit peptides for chloroplastic localization (Mustroph et al., 2007).

A remarkable feature of ATP‐PFK is its reversible inactivation through *reduction* (Hess et al., 2021; Yoshida & Hisabori, 2021). In Arabidopsis chloroplastic PFK5, the formation of an intramolecular disulfide bond between the N‐terminal cysteine residues 152 and 157 is a determinant for its redox regulation (Yoshida & Hisabori, 2021). This reduction is selectively catalyzed by stromal thioredoxin (Trx)‐*f* (Hess et al., 2021; Yoshida & Hisabori, 2021). Conversely, PFK5 can be oxidized and subsequently activated through the reconstituted Trx‐like2 (TrxL2)/2‐Cys peroxiredoxin (2CP) pathway (Yoshida & Hisabori, 2021). In the context of chloroplastic glycolysis, PFK5 catalyzes the reverse reaction of the Calvin–Benson cycle enzyme fructose‐1,6‐bisphosphatase (FBPase), consuming ATP. Interestingly, both PFK5 and FBPase exhibit a preference for Trx‐*f* as the Cys‐switch operator for both their deactivation (Yoshida & Hisabori, 2021) and activation (Balmer & Schürmann, 2001). The finely orchestrated interplay between these two enzymes ensures efficient metabolic regulation, preventing carbon loss by futile cycling (Yoshida & Hisabori, 2021).

### Aldolase

Aldolase (ALD; EC 4.1.2.13) catalyzes the reversible cleavage of F1,6P into d‐glyceraldehyde 3‐phosphate (G3P) and dihydroxyacetone phosphate (DHAP) during glycolysis and the reverse reaction during gluconeogenesis (Figure 1). Additionally, ALD plays a role in the Calvin–Benson cycle (Flechner et al., 1999; Gross et al., 1999). In Arabidopsis, eight ALD isoforms were identified. Three of these (ALD1‐3) are localized in the plastids, while the remaining five (ALD4‐8) are found in the cytosol (Lu et al., 2012).

Cytosolic ALD (cALD) has been identified as a target of *S‐glutathionylation* and *S‐nitrosylation in vivo* (Dixon et al., 2005; Ito et al., 2003; Lindermayr et al., 2005). *In vitro* experiments have confirmed these modifications upon incubation of cALD with glutathione disulfide (GSSG, reduced glutathione) and *S*‐nitrosoglutathione (GSNO), respectively (van der Linde et al., 2011). *S*‐glutathionylation leads to partial and reversible inactivation of the enzyme. This inactivation can be reversed by dithiothreitol (DTT) or the cytosolic NADPH‐dependent thioredoxin reductase (NTR) system (Marchand et al., 2006; van der Linde et al., 2011; Yamazaki et al., 2004). *S*‐nitrosylation results in total and irreversible inactivation of the enzyme, even at low concentrations of GSNO. F1,6P counteracts the inactivation of cALD caused by GSSG and GSNO, likely by inducing a conformational change that makes the cysteine residues inaccessible to the oxidizing agents. This suggests that the substrate may play a regulatory role in modulating cALD activity.

Mass spectrometry analysis has identified specific cysteine residues involved in these modifications: Cys68 is a site for *S*‐glutathionylation, while Cys173 undergoes both *S*‐glutathionylation and *S*‐nitrosylation (van der Linde et al., 2011). However, enzyme mutants lacking one or both of these cysteines still showed reduced inactivation by GSNO, but to a lesser extent. Furthermore, these mutants exhibited faster and more complete inactivation when exposed to oxidizing reagents such as GSSG and diamide, suggesting that other cysteine residues may also be sensitive to these reagents (van der Linde et al., 2011). The inactivation of cALD by *S‐glutathionylation* and *S‐nitrosylation* can slow down glycolysis and the TCA cycle, leading to impeded mitochondrial metabolism and preventing the generation of deleterious ROS under oxidative stress (Cabiscol et al., 2000).

### Triosephosphate isomerase

Triosephosphate isomerase (TPI; EC 5.3.1.1) catalyzes the reversible isomerization of DHAP to G3P (Figure 1). This enzyme exhibits interesting evolutionary adaptations across different plant species. Land plants possess two TPI isoforms: a cytosolic one (cTPI) involved in glycolysis and a chloroplastic one (pTPI) involved in the Calvin–Benson cycle (Kurzok & Feierabend, 1984). In contrast, green algae contain only one pTPI because the initial steps of glycolysis, up to the G3P‐DHAP interconversion, occur exclusively in the chloroplast in these organisms (Zaffagnini et al., 2014).

In Arabidopsis, cTPI activity is inhibited by *S‐glutathionylation* (Dumont et al., 2016; Ito et al., 2003). The presence of GSSG reduces cTPI activity, but this inhibition can be reversed *in vitro* by adding reduced glutathione (GSH). Additionally, glutaredoxins C1 (GRXC1) and C2 (GRXC2) can restore cTPI activity through deglutathionylation (Dumont et al., 2016). Two cysteine residues in cTPI, Cys127 and Cys218, have been identified as *S*‐glutathionylation sites. Mutation studies have revealed that changing Cys127 to serine causes a significant decrease in enzyme activity, while the mutation of Cys218 to serine does not affect the activity (Dumont et al., 2016).

In the green alga *Chlamydomonas reinhardtii*, the pTPI can also undergo *S*‐glutathionylation. However, in this case, the modification leads to only a slight decrease in protein activity (Zaffagnini et al., 2014). This difference in the impact of *S*‐glutathionylation between species underscores the diverse regulatory mechanisms that have evolved to control TPI activity in different photosynthetic organisms.

### Non‐phosphorylating glyceraldehyde‐3‐phosphate dehydrogenase

In the cytosolic glycolytic pathway, triose phosphates partitioning represents a critical metabolic step where G3P can be directed toward either ATP or NADPH production. Non‐phosphorylating glyceraldehyde‐3‐phosphate dehydrogenase (npGAPDH; EC 1.2.1.9) is an unconventional cytosolic glycolytic enzyme found in plant cells that catalyzes the irreversible oxidation of G3P to 3‐phosphoglycerate (3PGA), using NADP as a cofactor (Figure 1).

The regulation of npGAPDH in heterotrophic tissues involves a complex interplay of posttranslational modifications and protein–protein interactions. A key regulatory mechanism involves the *phosphorylation* of npGAPDH by a Sucrose non‐Fermenting 1‐Related protein Kinase 1 (SnRK1) (Bustos & Iglesias, 2002; Piattoni et al., 2011). SnRK1 plays a pivotal role in modulating the TOR pathway, integrating energy status with cellular processes to optimize growth and stress responses (Baena‐González & Hanson, 2017; Margalha et al., 2019). Following phosphorylation at Ser404 in wheat, npGAPDH interacts with 14‐3‐3 regulatory proteins to form a complex (Bustos & Iglesias, 2002, 2005). This complex formation results in reduced npGAPDH activity and increased susceptibility to inhibition by adenylates and pyrophosphate (PP_i_) (Piattoni et al., 2011). This regulatory mechanism serves as a control of triose phosphate utilization for NADPH production, particularly in non‐photosynthetic cells where it was suggested that high levels of ATP and/or PPi indicate elevated cytosolic energy content (Bustos & Iglesias, 2002; Piattoni et al., 2011).

*Redox* changes also play a role in modulating npGAPDH activity. Interestingly, when wheat and maize seedlings are exposed to oxidative stress induced by methyl viologen, npGAPDH activity increased by up to twofold, possibly due to enhanced enzyme stability (Bustos et al., 2008). However, *in vitro* studies have shown that direct exposure to oxidants can lead to varying degrees of npGAPDH inactivation that can be reversed by the addition of Trx‐*h* (Piattoni et al., 2013). The redox sensitivity of npGAPDH is likely linked to its structural features. In wheat npGAPDH, two strictly conserved cysteine residues, Cys271 and Cys422, which are absent in prokaryotic proteins, have the potential to form a disulfide bond. This redox‐sensitive structural element may serve as a protective mechanism, preserving the catalytic pocket from oxidation and potentially modulating substrate binding (Bustos & Iglesias, 2005).

By integrating signals from energy status (through phosphorylation and protein complex formation) and redox state (through cysteine modifications), npGAPDH can tune the balance between energy and reducing power provision in cytosolic glycolysis.

### Phosphorylating glyceraldehyde‐3‐phosphate dehydrogenase

Phosphorylating glyceraldehyde‐3‐phosphate dehydrogenase (GAPDH; EC 1.2.1.12) is a glycolytic enzyme that catalyzes the conversion of G3P to 1,3‐bisphosphoglycerate (1,3BPG), using NAD as a cofactor (Figure 1). In plants, glycolytic GAPDH exists in multiple isoforms distributed in cytosol (GAPC1 and GAPC2) and plastids (GAPCP1 and GAPCP2) (Petersen et al., 2003). Beyond its primary metabolic role, cytosolic GAPDH has gained recognition for its moonlighting functions, including acting as a transcriptional regulator (Kim et al., 2020; Peralta et al., 2016; Zaffagnini, Fermani, et al., 2013; Zhang et al., 2017).

Wheat recombinant GAPDH undergoes *phosphorylation* at Ser205 by SnRK1 from heterotrophic tissues (Piattoni et al., 2017). The S205D mutant enzyme, which mimics the phosphorylated form, exhibits a significant reduction in activity while maintaining a similar affinity for substrates. This phosphorylation of cytosolic GAPDH has also been observed *in vivo* in developing wheat seeds (Piattoni et al., 2017). As wheat seeds mature, a gradual increase in GAPDH activity is observed. Interestingly, protein levels and phosphorylation status show little variation throughout this process. However, the levels of phosphorylated GAPDH peaked when the seed transitioned its metabolism to primarily support the synthesis and accumulation of carbon reserves (Piattoni et al., 2017). This timing suggests a potential regulatory mechanism linking GAPDH phosphorylation to the critical metabolic shift toward storage product synthesis in developing seeds.

In Arabidopsis, the interaction of GAPC1 with the ubiquitin E3 ligase Seven In Absentia Like 7 (SINAL7) via Lys23 enhances its catalytic activity, potentially increasing the flux of glycolysis (Peralta et al., 2016). In the presence of the complete E1‐E2‐E3 modifier complex, SINAL7 *mono‐ubiquitinates* GAPC1 at Lys76, abolishing catalytic activity and triggering its translocation to the nucleus, where it acts as a transcriptional activator of several glycolytic genes, including hexokinase, PFK, and PK (Peralta et al., 2016; Zhang et al., 2017).

Arabidopsis GAPC2 is *acetylated* at multiple lysine residues (130, 216, 220, and 255) when expressed in *Escherichia coli*, and removal of acetylation by human SIRT3 increases the enzymatic activity by threefold (Finkemeier et al., 2011). In rice, acetylation of cytosolic GAPDH1 on several lysine residues promotes its translocation from the cytosol to the nucleus, where its moonlighting activity transcriptionally activates glycolysis (Zhang et al., 2017). Simultaneous mutation of Lys57, Lys74, and Lys217 or incubation with rice SRT1 enhances enzymatic activity and decreases nuclear accumulation (Zhang et al., 2017).

Thiol modifications, specifically *S‐nitrosylation* and *S‐glutathionylation*, reversibly inhibit GAPC1 and GAPC2 by targeting two conserved cysteine residues; one of these residues is involved in catalysis, while the other participates in a proton relay mechanism (Holtgrefe et al., 2008; Zaffagnini, Fermani, et al., 2013; Zaffagnini, Morisse, et al., 2013). When GAPDH is oxidized, glycolysis is modulated to enhance NADPH production through two mechanisms: the action of npGAPDH and the diversion of metabolic flux into the pentose phosphate pathway. Beyond affecting enzymatic activity, oxidative thiol modifications are also essential for protein–protein interactions and nuclear relocalization of GAPDH (Vescovi et al., 2013). *In vivo* studies using Arabidopsis cell cultures have demonstrated that *S*‐glutathionylation leads to a significant decrease in GAPDH enzymatic activity (Dixon et al., 2005). When GAPDH is treated with GSH followed by either H_2_O_2_ or GSSG, its activity can be restored by the subsequent addition of dithiothreitol (DTT). However, treatment with H_2_O_2_ alone results in an irreversible modification, permanently inhibiting the enzyme (Zaffagnini et al., 2012). Increased *S*‐nitrosylation has been observed in tobacco GAPCa and GAPCb under salt stress (Wawer et al., 2010).

*S‐sulfhydration* of Arabidopsis recombinant GAPC1 enhances its activity, while treatment with DTT reduces it (Aroca et al., 2015). This PTM also influences the nuclear localization of GAPC1 and GAPC2. In wild‐type plants, hydrogen sulfide treatment increases the nuclear accumulation of these enzymes. Conversely, mutants deficient in cytosolic sulfide production exhibit reduced nuclear localization of GAPC1 and GAPC2 (Aroca et al., 2017). Specifically, *S*‐sulfhydration of GAPC1 occurs specifically at Cys160 (Aroca et al., 2017).

The diverse posttranslational modifications of cytosolic GAPDH in plants orchestrate a complex regulatory network. These modifications tune the enzymatic activity, subcellular localization, and moonlighting roles, establishing it as a pivotal metabolic sensor and signaling hub. This positioning influences carbon metabolism, energy conversion, cell death, and stress response pathways (Hildebrandt et al., 2015; Peralta et al., 2016; Piattoni et al., 2017; Zhang et al., 2017).

### 2,3‐Bisphosphoglycerate‐independent phosphoglycerate mutase

Phosphoglycerate mutase (PGM) catalyzes the reversible isomerization of 3PGA to 2‐phosphoglycerate (2PGA) (Figure 1). Two distinct classes of PGM exist, differentiated by their cofactor requirements: 2,3‐bisphosphoglycerate‐dependent PGM (dPGM, EC 5.4.2.11) and 2,3‐bisphosphoglycerate‐independent PGM (iPGM, EC 5.4.2.12) (Jedrzejas, 2000). In plants, iPGM is specifically responsible for PGA isomerization (Fothergill‐Gilmore & Watson, 1989).

Arabidopsis possesses one plastidial (iPGM1) and two cytosolic (iPGM2 and iPGM3) iPGM isoforms (Zhao & Assmann, 2011). Recent phosphoproteomics studies have revealed that both cytosolic isoforms undergo *phosphorylation* at a serine residue crucial for catalysis, Ser80 in iPGM1 and Ser82 in iPGM2 (Duminil et al., 2021). Notably, substituting Ser82 with either alanine or aspartate completely abolishes the 3PGA‐dependent activity. Previous research on non‐plant iPGM has shown that catalysis involves a transient phosphoserine intermediate that carries the phosphate group during the isomerization reaction (Mercaldi et al., 2012; Roychowdhury et al., 2015). This suggests that the phosphorylation observed at Ser80/82 is more likely a consequence of the isomerization reaction itself, rather than a PTM mediated by a kinase to regulate iPGM activity. Nevertheless, the transient nature of Ser82 phosphorylation provides a unique opportunity to study steady‐state catalysis under different conditions, such as light and dark cycles. Interestingly, in Arabidopsis leaves, the phosphorylation levels of Ser80/82 are found to be higher in light conditions compared to dark conditions (Duminil et al., 2021). This observation hints at the potential diurnal regulation of iPGM activity, which may be linked to changes in metabolic demands.

### Enolase

Enolase (ENO; EC 4.2.1.11) catalyzes the dehydration of 2PGA to phosphoenolpyruvate (PEP), the immediate precursor of pyruvate (Figure 1) (Balmer et al., 2003). In Arabidopsis, three enolase genes are present: *ENO1*, *ENO2*, and *ENO3*. ENO2 is the primary active cytosolic isoform (Andriotis et al., 2010) and has been identified as a target for various redox modifications (Dumont & Rivoal, 2019).

Recent research has revealed a novel role for ENO2 in cold stress response. It was demonstrated that cold‐induced H_2_O_2_ *sulfenylates* ENO2 at Cys408, promoting its oligomerization (Liu et al., 2022). This modification enhances ENO2's nuclear import and its binding to the promoter of C‐repeat/DRE binding factor1 (CBF1), a crucial transcription factor in plant cold signaling. The resulting activation of CBF1 expression initiates a cascade of cold response genes, establishing a direct link between H_2_O_2_ signaling and plant freezing tolerance through ENO2 activity modulation.

Earlier studies showed that diamide, a thiol‐specific oxidant promoting protein *S‐glutathionylation*, increases ENO2 activity in Arabidopsis extracts (Anderson et al., 1998). This effect was hypothesized to result from a disulfide bond formation between Cys313 and Cys339. However, the precise mechanisms and physiological implications of these redox modifications on ENO2 regulation remain to be fully elucidated.

### Pyruvate kinase

Pyruvate kinase (PK; EC 2.7.1.40) catalyzes the irreversible final step of glycolysis, converting PEP to pyruvate while simultaneously phosphorylating ADP to ATP (Figure 1). It exists as both cytosolic (cPK) and plastidial (pPK) isoforms (Ambasht & Kayastha, 2002). As a critical enzyme at the intersection point of several metabolic pathways (Figure 1), PK regulates the utilization of PEP for diverse metabolic routes, including lipid biosynthesis, protein biosynthesis, or energy conversion. Consequently, PK activity and protein levels are tightly regulated through various mechanisms, including PTMs.

In soybean seeds, cPK activity is regulated by partial *C‐terminal truncation*, leading to the generation of a 51 kDa polypeptide from the 55 kDa cPK subunit (Tang et al., 2003). Extracts from 7‐week‐old seeds, which are enriched in the 51 kDa polypeptide, exhibit cPK activation in the presence of aspartate. Conversely, extracts from 2‐week‐old seeds, mainly containing the 55 kDa polypeptide, show slight inhibition of cPK activity. These observations suggest that C‐terminal truncation might modulate the activation of the 55 kDa cPK (Tang et al., 2003). It is also speculated that the shorter cPK has increased stability, as its content rises during seed development.

In soybean seeds cPK, *phosphorylation* events at the Ser220 and Ser407 primarily occur on N‐terminal truncated polypeptides (Tang et al., 2003). Similarly, a cotton cPK can be phosphorylated at Ser215 and Ser402 (Zhang & Liu, 2017). Phosphorylation of cPK at Ser215 inhibited its enzyme activity, whereas phosphorylation at both serine sites promotes its degradation. In both cases, phosphorylation is a prerequisite for targeting cPK for *ubiquitination* (Tang et al., 2003; Zhang & Liu, 2017). These processes contribute to the degradation of cPK via the ubiquitin/26S proteasome pathway (Tang et al., 2003). Degradation of PK by this system is enhanced during the first stages of cotton fiber elongation; therefore, reduced activity of the enzyme likely favors fast cell growth (Zhang & Liu, 2017).

### Pyruvate, orthophosphate dikinase

Pyruvate, orthophosphate dikinase (PPDK; EC 2.7.9.1) catalyzes the reversible ATP‐dependent interconversion of PEP and pyruvate in both the chloroplasts and cytosol (Figure 1). Chloroplastic PPDK plays roles in non‐photosynthetic metabolism and C4 photosynthesis (Chastain et al., 2006). The non‐photosynthetic role of PPDK is to supplement the stromal pool of PEP for aromatic amino acid biosynthesis via the shikimate pathway (Chastain & Chollet, 2003). Similar to C4‐photosynthetic PPDK, plastidial non‐photosynthetic PPDK is also reversibly inactivated through phosphorylation of a threonine residue (Balparda et al., 2023).

PPDK reaction in the cytosol is crucial in gluconeogenesis and is considered to reverse the action of pyruvate kinase (Yu et al., 2021). In developing rice seeds, PPDK is highly abundant in the cytosol of endosperm cells, where it supports biosynthetic processes by providing pyruvate and likely contributing to ATP synthesis in oxygen‐depleted tissues. As rice seed development progresses, PPDK undergoes reversible inactivation through *phosphorylation* of a threonine residue and experiences a reduction in its abundance via protein degradation (Chastain et al., 2006). This phosphorylation is catalyzed by a PPDK regulatory protein (RP), which functions as a protein kinase in the cytosol (Chastain et al., 2008). The phosphorylation and activation status of cytosolic PPDK appear to be linked to cellular ADP levels as a mechanism of regulation.

### Phosphoenolpyruvate carboxylase

Glycolytic PEP carboxylase (PEPC; EC 4.1.1.31) is located in the cytosol and catalyzes the irreversible β‐carboxylation of PEP in the presence of ${\mathrm{HCO}}_{3}^{-}$ to produce oxaloacetate (OAA) and inorganic phosphate (Figure 1). As an anaplerotic enzyme in carbohydrate metabolism, PEPC supplies carbon skeletons for the TCA cycle (Figure 1). Given its position at a metabolic branchpoint, changes in carbon partitioning are crucial to synchronize cell metabolism with the environment, making PEPC a central point for the regulation of cellular energy metabolism. PEPC is also involved in carbon concentrating mechanisms in C4 and Crassulacean Acid Metabolism (CAM) plants (Balparda et al., 2023).

Similar to C4 and CAM PEPC, glycolytic PEPC is allosterically activated by glucose 6‐phosphate and inhibited by malate (Balparda et al., 2023). Additionally, the enzyme undergoes *phosphorylation* at a conserved serine residue in its N‐terminus, which reduces malate inhibition and increases glucose 6‐phosphate activation (Duff & Chollet, 1995; Tripodi et al., 2005). A specific Ca^2+^‐independent PEPC kinase is responsible for phosphorylating PEPC, while it is dephosphorylated by a protein phosphatase type‐2A (Law & Plaxton, 1997; Xu et al., 2003).

PEPC is also *mono‐ubiquitinylated* in heterotrophic tissues of different species (Ruiz‐Ballesta et al., 2014; Ruiz‐Ballesta et al., 2016; Shane et al., 2013; Uhrig et al., 2008). For example, in developing castor oil seeds, Lys628 was identified as the site of mono‐ubiquitination, which interferes with PEP binding to the adjacent catalytic domain while enhancing its sensitivity to inhibition by malate and aspartate (Uhrig et al., 2008). Consequently, mono‐ubiquitination of PEPC in developing castor oil seeds is inhibitory, decreasing the affinity for PEP and increasing sensitivity to allosteric inhibitors. Similarly, the mono‐ubiquitination of PEPC in developing proteoid roots of *Hakea prostrata* reduces the enzyme's activity (Shane et al., 2013). In sorghum PEPC, mono‐ubiquitination occurs at conserved Lys630 or Lys624 on different isoforms (Ruiz‐Ballesta et al., 2016). Notably, phosphorylation and mono‐ubiquitination are not mutually exclusive in sorghum PEPC isoforms, where both modifications occur simultaneously in specific stages of development and germination (Ruiz‐Ballesta et al., 2016). These results suggest that a combination of both PTMs may serve to fine‐tune the precise kinetic and regulatory properties for each PTPC isoenzyme at each specific stage of development and germination.

### Phosphoenolpyruvate carboxykinase

Phosphoenolpyruvate carboxykinase (PEPCK; EC 4.1.1.49) catalyzes the ATP‐dependent decarboxylation of OAA derived from the glyoxylate cycle or exported from the mitochondria into PEP (Figure 1). PEPCK plays a crucial role in fat‐storing seedlings by initiating the gluconeogenic conversion of storage lipids into sucrose. Additionally, PEPCK plays a pivotal role in the CO_2_‐concentrating mechanism of C4 and CAM plants during photosynthesis (Bailey et al., 2007). As PEPCK is exclusively located in the cytosol (Watanabe et al., 1984), its activity requires tight regulation as its combined action with PEPC would lead to a futile cycle between PEP and OAA, resulting in ATP hydrolysis without net metabolic gain.

*Phosphorylation* is a conserved regulatory principle of PEPCK in many plants (Walker et al., 2016; Walker & Leegood, 1995, 1996), occurring at the N‐terminal extension of this enzyme. Sequencing analysis of plant PEPCK reveals that the N‐terminal extension typically contains one or two potential phosphorylation sites (Kim & Smith, 1994). Light/dark transitions play a significant role in the phosphorylation of PEPCK, with the enzyme being phosphorylated in the dark and dephosphorylated in the light (Walker & Leegood, 1995, 1996). Dephosphorylation effectively increases PEPCK activity by altering its affinity to substrates at a given ATP/ADP ratio. This regulatory principle allows the enzyme to efficiently respond to changing energy demands during different phases of the plant's life cycle (Walker et al., 2002).

### Cytosolic malate dehydrogenase

Malate dehydrogenase (MDH; EC 1.1.1.37) catalyzes the reversible conversion of malate into OAA, using NAD(P)^+^ or NAD(P)H as cofactors (Figure 1). Found in various subcellular compartments, MDH plays significant roles in plant energy metabolism and redox homeostasis between organellar compartments (Scheibe, 2004; Scheibe & Dietz, 2012; Selinski & Scheibe, 2019). Cytosolic MDH (cMDH) is involved in the malate/aspartate and malate/OAA shuttles, facilitating the indirect transfer of reducing equivalents between the cytosol and mitochondria (Scheibe, 2004). It also participates in gluconeogenesis by converting malate to OAA, which is then converted to PEP by PEPCK. In Arabidopsis, three cytosolic isoforms of MDH exist (cMDH1‐3), with cMDH1 exhibiting the highest transcript abundance (Liszka et al., 2020).

Under oxidizing conditions, cMDH1 undergoes cysteine *sulfenylation* and methionine *sulfoxidation*, leading to enzyme inactivation (Huang et al., 2018). This inactivation results from a conformational change caused by the formation of an intermolecular disulfide bond between Cys330 residues on monomeric proteins, resulting in homodimer formation. The sensitivity of cMDH1 to oxidation is also observed *in vivo*, as cMDH1 activity significantly decreases in mutants accumulating high levels of H_2_O_2_ and impaired in the Trx reduction systems (Huang et al., 2018). cMDH1 activity can be reactivated by adding the NTR/Trx‐*h3* system to plant extracts from these mutants, supporting a Trx‐dependent regulation of cMDH1 activity (Hara et al., 2006; Huang et al., 2018).

Oxidation of cMDH2 by diamine treatment leads to inactivation and dimerization through the formation of *disulfide bridges* (Liszka et al., 2020). In contrast, while cMDH3 also forms dimers and higher oligomers under oxidizing conditions, its activity is less affected. Glutathione protects both cMDH1 and cMDH2 from dimerization and inactivation. cMDH3, however, still dimerizes but does not form oligomers in the presence of glutathione. Additionally, oxidative stress can trigger the relocation of all three cMDH isoforms to the nucleus, with cMDH3 showing the most pronounced nuclear localization (Liszka et al., 2020). The unique C2–C2‐disulfide‐linked dimer of cMDH3 is, therefore, a promising candidate as a redox sensor, potentially assuming moonlighting functions during disturbances in energy metabolism. Nevertheless, all three cMDHs are candidates for integrating redox‐ and metabolic information and gene expression.

## REGULATION OF THE TCA CYCLE AND ASSOCIATED PROTEINS BY PTMs

The TCA cycle orchestrates the oxidation of acetyl‐CoA derived from various sources – including carbohydrates, lipids, and amino acids – to transfer electrons to NAD and FAD (Figure 1). However, the TCA cycle is not a static pathway. Instead, the flux mode can change flexibly to meet cellular needs not only for the provision of reducing power and ATP but also for the supply of carbon skeletons for amino acid, purine nucleotide, and secondary metabolite syntheses, as well as ammonia fixation. To fulfill such a variety of functions, TCA cycle enzymes can support a spectrum of different flux modes, both cyclic and non‐cyclic (Sweetlove et al., 2010).

In plants, regulation of the TCA cycle primarily involves at least three mechanisms: allosteric control, physical association, and PTMs. Among the various PTMs, redox regulation stands out as an important mechanism for modulating TCA cycle enzyme activity in plants. Most TCA cycle enzymes have been found to contain surface‐exposed cysteine thiols that are candidates to mediate redox regulation (Balmer et al., 2004; Nietzel et al., 2020; Yoshida et al., 2013). *O*‐type Trx proteins have been described as regulators of carbon flux through the TCA cycle by modulating enzyme activities. Indeed, Trx‐mediated activation and deactivation of TCA cycle enzymes may help to explain the noncyclic flux mode observed in illuminated leaves (Sweetlove et al., 2010), where there is an increased flux from citrate to glutamate biosynthesis and from malate to fumarate (Cheung et al., 2014; Daloso et al., 2015).

In addition to Trx‐mediated redox regulation, other PTMs are clearly involved in the regulation of TCA cycle enzymes. This section examines the effect of various PTMs on TCA cycle enzyme activities, highlighting our current understanding of several intricate control mechanisms that contribute to the flexibility and maintenance of the TCA cycle (Table 1).

**Table 1:** Summary of PTMs regulating components of plant glycolysis and TCA cycle

Metabolic pathway	Protein	PTMs	Functional effect	Reference
Glycolysis	Hexokinase (EC 2.7.1.1)	Phosphorylation	Catalytically inactive	Moore et al. (2003)
	ATP‐phosphofructokinase (EC 2.7.1.11)	Redox switch	Inactivation by reduction	Hess et al. (2021); Yoshida and Hisabori (2021)
	Aldolase (EC 4.1.2.13)	*S*‐glutathionylation	Partial and reversible inactivation	van der Linde et al. (2011)
		*S*‐nitrosylation	Total and irreversible inactivation	van der Linde et al. (2011)
	Triosephosphate isomerase (EC 5.3.1.1)	*S*‐glutathionylation	Inhibits enzyme activity	Dumont et al. (2016); Ito et al. (2003); Zaffagnini et al. (2014)
	Non‐phosphorylating glyceraldehyde‐3‐phosphate dehydrogenase (EC 1.2.1.9)	Phosphorylation	Reduced activity and increased susceptibility to inhibition by adenylates and PPi	Bustos and Iglesias (2002); Piattoni et al. (2011)
		Redox switch	Reversible inactivation by oxidation	Piattoni et al. (2013)
	Phosphorylating glyceraldehyde‐3‐P dehydrogenase (EC 1.2.1.12)	Mono‐ubiquitination	At Lys23 enhances its catalytic activity At Lys76 inactivation and prompts its translocation to the nucleus	Peralta et al. (2016); Zhang et al. (2017)
		Lys‐acetylation	The enzymatic activity increases upon deacetylation and increased nuclear accumulation	Finkemeier et al. (2011); Zhang et al. (2017)
		*S*‐glutathionylation	Reversible inhibition of enzyme activity	Holtgrefe et al. (2008)
		*S*‐nitrosylation	Reversible inhibition of enzyme activity and relocalization to nucleus	Vescovi et al. (2013); Zaffagnini, Morisse, et al. (2013)
		*S*‐sulfhydration	Enhancing its nuclear localization	Aroca et al. (2017)
		Phosphorylation	Reduction of its catalytic efficiency	Piattoni et al. (2017)
	2,3‐Biphosphoglycerate independent phosphoglycerate mutase (EC 5.4.2.12)	Phosphorylation	Essential for enzyme activity	Duminil et al. (2021)
	Enolase (EC 4.2.1.11)	Sulfenylation	Promotes its oligomerization and nuclear import	Liu et al. (2022)
		*S*‐glutathionylation	Increases activity	Anderson et al. (1998)
	Pyruvate kinase (EC 2.7.1.40)	C‐terminal truncation	Activates enzyme and increases its stability	Tang et al. (2003)
		Phosphorylation	Inhibits enzyme activity and promote its degradation by ubiquitination	Zhang and Liu (2017)
		Ubiquitination	Degradation of the protein	Tang et al. (2003); Zhang and Liu (2017)
	Pyruvate, orthophosphate dikinase (EC 2.7.9.1)	Phosphorylation	Inactivates the enzyme	Chastain et al. (2006)
	Phosphoenolpyruvate carboxylase (EC 4.1.1.31)	Phosphorylation	Reduces malate inhibition and increases glucose 6‐phosphate activation	Duff and Chollet (1995); Tripodi et al. (2005)
		Mono‐ubiquitination	Decrease activity	Shane et al. (2013); Uhrig et al. (2008)
	Phosphoenolpyruvate carboxykinase (EC 4.1.1.49)	Phosphorylation	Dephosphorylation increases its activity	Walker et al. (2002)
	Cytosolic malate dehydrogenase (EC 1.1.1.37)	Redox switch Sulfenylation Sulfoxidation	Reversible inactivation by oxidation	Hara et al. (2006); Huang et al. (2018); Liszka et al. (2020)
TCA cycle	Pyruvate dehydrogenase complex (EC 1.2.1.104)	Phosphorylation	Inactivates the enzyme	Rubin and Randall (1977); Thelen et al. (2000)
	Citrate synthase (E.C. 2.3.3.1)	Redox switch	Inactivation by oxidation	Schmidtmann et al. (2014)
		*S*‐nitrosylation	Increases enzyme activity	Wei et al. (2022)
	Aconitase (EC 4.2.1.3)	Redox switch	Inhibition by oxidation	Daloso et al. (2015); Obata et al. (2011); Verniquet et al. (1991)
		*S*‐nitrosylation	Inactivation of the enzyme	Gupta et al. (2012)
	NAD^+^‐dependent isocitrate dehydrogenase (EC 1.1.1.41)	Redox switch	Inactivation by oxidation	Yoshida and Hisabori (2014)
	Succinate dehydrogenase (EC 1.3.5.1)	Redox switch	Inactivation by Trx system	Daloso et al. (2015)
	Fumarase (EC 4.2.1.2)	Redox switch	Inactivation by oxidation	Daloso et al. (2015); Zubimendi et al. (2018)
	Mitochondrial malate dehydrogenase (EC 1.1.1.37)	Lys‐acetylation	Leads to either activation or inhibition of mMDH	Balparda et al. (2022)
	NAD^+^‐malic enzyme (EC 39.1.1.1)	Redox switch	Activation by reduction	Nietzel et al. (2020)

PTM, posttranslational modification; TCA, tricarboxylic acid.

### Pyruvate dehydrogenase complex

The mitochondrial pyruvate dehydrogenase complex (PDC; EC 1.2.1.104) catalyzes the oxidative decarboxylation of pyruvate, producing acetyl‐CoA and NADH (Figure 1). This reaction serves as a key entry point for carbon into the TCA cycle, linking glycolysis with aerobic respiration. PDC is a multienzyme complex composed of three catalytic components: pyruvate dehydrogenase (PDH; EC 1.2.4.1), dihydrolipoyl acetyltransferase (DLAT, EC 2.3.1.12) and dihydrolipoyl dehydrogenase (DLD; EC 1.8.1.4) (Tovar‐Méndez et al., 2003). Plant cells contain two distinct PDCs: one located in the mitochondria, which feeds acetyl‐CoA into the TCA cycle for energy conversion and can be translocated to the nucleus in the presence of ethylene (Shao et al., 2024); a second PDC is found in plastids, which provides acetyl‐CoA and NADH for de novo fatty acid biosynthesis (Krämer & Kunz, 2021).

To optimize carbon resources, PDC activity is tightly regulated, especially in high‐ATP environments such as during active photosynthesis in daylight hours (Elsässer et al., 2020; Gardeström & Igamberdiev, 2016; Santarius & Heber, 1965; Vera‐Vives et al., 2024). This regulation involves complex metabolite‐mediated regulation (Bocobza et al., 2013; Tovar‐Méndez et al., 2003) and reversible *phosphorylation* (Rubin & Randall, 1977). However, the relative contributions of phosphorylation and the various metabolite effectors to PDC regulation remain to be fully elucidated.

In the light, PDC activity is inhibited through two primary mechanisms: phosphorylation at multiple serine sites on the PDH subunit (Giese et al., 2023; Thelen et al., 2000) and product inhibition by NADH and acetyl‐CoA (Budde et al., 1991). Ammonium and glyoxylate also act as inhibitors, while pyruvate and thiamine pyrophosphate are activators of PDC (Bocobza et al., 2013; Plaxton & Podestá, 2006; Tovar‐Méndez et al., 2003). The phosphorylation of PDC is catalyzed by a PDC‐specific pyruvate dehydrogenase kinase (PDK; EC 2.7.11.2), a histidine kinase that specifically targets serine residues of the PDH subunit (Schuller & Randall, 1990; Thelen et al., 2000). PDK is inhibited by ADP and pyruvate, providing a feedback mechanism for fine‐tuning PDC regulation. In the darkness, PDC is activated through dephosphorylation primarily mediated by two phosphatases: PDC phosphatase (PDP; EC 3.1.3.43) (Tovar‐Méndez et al., 2003) and the type 2C protein phosphatase 2C 63 (PP2c63) (Zhang et al., 2021). Additionally, the SIT4 phosphatase‐associated protein (Sal2) regulates PDC activity in conjunction with PP2c6, exhibiting slightly overlapping activities (Zhang et al., 2021). The importance of these regulatory mechanisms is highlighted by the phenotypes of mutants lacking both PP2c63 and Sal2, which exhibit reduced leaf number and overall growth (Zhang et al., 2021). This suggests that these phosphatases likely act on multiple TCA cycle enzymes with partial redundancy, underscoring the complex interplay between PDC regulation and plant growth and development.

The phosphorylation of PDC shifts the carbon flux between chloroplasts and mitochondria and may favor the utilization of pyruvate in biosynthetic pathways such as fatty acid synthesis by preventing unnecessary oxidation of pyruvate in mitochondria (Gemel & Randall, 1992; Rawsthorne, 2002).

### Citrate synthase

Citrate synthase (CS; E.C. 2.3.3.1) catalyzes the initial reaction of the TCA cycle, condensing an acetyl group with OAA to form citrate (Figure 1). The Arabidopsis genome encodes five CS isoforms: two localized to mitochondria (CS4 and CS5), which are involved in the TCA cycle, and three to peroxisome (CS1–3), which participate in the glyoxylate cycle (Pracharoenwattana et al., 2005). Of the mitochondrial isoforms, CS4 is more abundant than CS5. Notably, mitochondrial CS expression is restricted to green tissue (Schmidtmann et al., 2014).

Arabidopsis CS4 is redox‐regulated. The enzyme's activity is changed through the reversible *oxidation* of specific cysteine residues, which form inter‐ and intramolecular disulfide bonds. Oxidant treatment with diamide and H_2_O_2_ inactivate the enzyme by promoting the formation of these disulfide bonds. Conversely, reductant treatment with dithiothreitol (DTT) and reduced Trx enhance enzyme activity by breaking these disulfide bridges (Schmidtmann et al., 2014; Stevens et al., 1997). Site‐directed mutagenesis studies of all cysteine residues in CS4 have revealed that Cys108 and Cys325 are critical for CS activity and contribute partially to the enzyme's redox sensitivity (Schmidtmann et al., 2014). Additionally, Cys365 and Cys467, while less important for overall activity, also contribute to the enzyme's redox sensitivity.

In tomato seedlings, CS activity is modulated under stress conditions. Salt stress elevates CS activity through *S‐nitrosylation* at Cys296, a process mediated by increased endogenous NO levels in the presence of GSNO (Wei et al., 2022). Interestingly, salt stress alone does not induce *S*‐nitrosylation to CS, suggesting that the relationship between environmental stress, nitric oxide signaling, and CS regulation is complex and warrants further investigation.

### Aconitase

Aconitase (ACO; EC 4.2.1.3) catalyzes the reversible isomerization of citrate to isocitrate (Figure 1) via cis‐aconitate as an intermediate (Peyret et al., 1995). In Arabidopsis, three nuclear‐encoded ACO isoforms are present, localized in both mitochondria and cytosol (Bernard et al., 2009; Hooks et al., 2014) (Heazlewood et al., 2004; Kruft et al., 2001; Millar et al., 2001). The active form of ACO is an iron–sulfur protein containing a 4Fe‐4S cluster coordinated by three cysteine residues (Peyret et al., 1995).

ACO is *redox‐sensitive* (Nietzel et al., 2020; Obata et al., 2011; Verniquet et al., 1991). Oxidation of the Fe–S cluster leads to the loss of the labile Fe atom (3Fe–4S), resulting in enzyme inactivation. In potatoes, H_2_O_2_ inhibits ACO activity both *in vivo* and *in vitro*, an effect partially mitigated by citrate addition (Verniquet et al., 1991). Similarly, Arabidopsis seedlings show significantly reduced ACO activity when treated with menadione (Lehmann et al., 2009). While ACO's amino acid sequence suggests potential regulation by Trx (da Fonseca‐Pereira et al., 2021), its activity is unexpectedly upregulated in Trx‐*o1* (*trxo1*) and NTR (*ntra ntrb*) mutants. However, the addition of recombinant Trx‐*o1* to wild‐type mitochondrial extracts does not affect enzymatic activity (Daloso et al., 2015). Moreover, NO inactivates ACO, leading to a marked citrate accumulation under hypoxic stress in Arabidopsis (Gupta et al., 2012). The precise mechanisms of redox regulation on ACO activity remain to be elucidated.

### Isocitrate dehydrogenase

In mitochondria, the oxidative decarboxylation of isocitrate to 2‐oxoglutarate is catalyzed by two independent enzymes: NADP^+^‐dependent isocitrate dehydrogenase (ICDH; EC 1.1.1.42) and NAD^+^‐dependent isocitrate dehydrogenase (IDH; EC 1.1.1.41) (Figure 1). The regulation of flux partitioning between IDH and ICDH remains an intriguing and unresolved question in central plant metabolism, with profound implications on the regulation of NAD‐ and NADP‐linked processes in the mitochondrial matrix. In Arabidopsis, only one gene encodes for mitochondrial ICDH (Niazi et al., 2019), while there are five genes encoding IDH subunits. IDH functions as a heterodimer, consisting of a regulatory (IDH‐r; IDH1, IDH2, and IDH3) and a catalytic (IDH‐c; IDH5 and IDH6) subunit (Lemaitre & Hodges, 2006).

**Figure 2:**
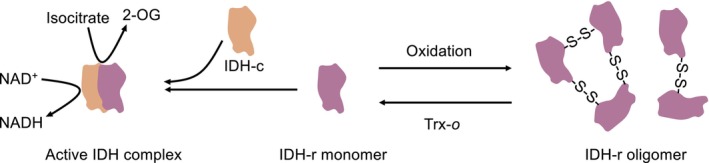
Model of IDH redox regulation. IDH‐r monomer is essential for forming an active IDH complex with IDH‐c. IDH‐r can be oxidized and form oligomers via intermolecular disulfide bonds. These oligomers can be reduced by Trx‐*o* and change back to monomers. 2‐OG, 2‐oxoglutarate; IDH‐c, NAD^+^‐dependent isocitrate dehydrogenase catalytic subunits; IDH‐r, NAD^+^‐dependent isocitrate dehydrogenase regulatory subunits. Adapted from Yoshida and Hisabori (2014).

While no PTMs have been identified for the mitochondrial ICDH to date, IDH was found to be *redox‐sensitive* (Figure 2). IDH is reversibly inactivated by oxidation and forms oligomers via intermolecular disulfide bonds between Cys128 and Cys216 of the IDH‐r subunit (Yoshida & Hisabori, 2014). Oxidized IDH‐*r* can be partially reactivated by reduced Trx‐*o*, which catalyzes disulfide reduction. While the evidence for IDH redox regulation relies on *in vitro* assays, future research will need to reveal whether IDH‐r homo‐oligomers are formed in situ under the physiological redox environments and in the presence of other matrix proteins (Figure 2).

### Succinate dehydrogenase

Succinate dehydrogenase (SDH; EC 1.3.5.1) is located in the inner membrane of mitochondria, where it plays a pivotal role in cellular respiration by serving as succinate: ubiquinone oxidoreductase, also known as complex II of the electron transport chain. SDH catalyzes the oxidation of succinate to fumarate, coupling this reaction to the reduction of ubiquinone. The enzyme contains a covalently bound FAD cofactor, which is reduced to FADH2 during the oxidation of succinate (Figure 1). The electrons from FADH2 are then transferred through a series of iron–sulfur clusters within the enzyme complex to ultimately reduce ubiquinone (UQ) to ubiquinol (UQH2). Thus, SDH links the TCA cycle with the electron transport system. In Arabidopsis, SDH is a heterooctameric complex composed of four catalytic subunits (SDH5–8) and four additional subunits (SDH1–4), which are involved in stabilizing the structure and possibly contributing to regulatory functions (Huang et al., 2019).

Recent studies have suggested that SDH activity is regulated by *redox mechanisms*. For instance, SDH activity is elevated in Arabidopsis mutants such as *trxo1* and *ntra ntrb*, indicating a potential link between redox state and enzyme function. Furthermore, the addition of recombinant Trx‐*o1* and Trx‐*h2* proteins to mitochondrial extracts has been shown to deactivate SDH *in vitro*, supporting the hypothesis that mitochondrial *o*‐type Trxs play a role in the *in vivo* regulation of SDH activity (Daloso et al., 2015). A mitochondrial redox‐proteomic study, which utilized citrate feeding to promote endogenous NADPH production within the mitochondrial matrix to drive the reduction of matrix‐exposed cysteine redox switches, identified six operational thiol switches on SDH1, SDH2, and SDH5 (Nietzel et al., 2020).

### Fumarase

Fumarase (FUM; EC 4.2.1.2) catalyzes the reversible hydration of fumarate to malate in the TCA cycle (Figure 1) (Mann & Woolf, 1930; Woods et al., 1988). In most organisms, including animals, yeast, and some plants such as *Acer pseudoplatanus*, a single gene encodes both cytosolic and mitochondrial FUM isoforms (Gout et al., 1993; Sass et al., 2001; Stein et al., 1994). Arabidopsis, however, possesses two distinct FUM genes: FUM1 encoding the mitochondrial form and FUM2 encoding the cytosolic form (Pracharoenwattana et al., 2010).

The activity of FUM is influenced by *redox* regulation. FUM activity is inhibited in *Arabidopsis* seedlings following menadione treatment, which leads to pronounced oxidation of the glutathione pool and possibly other cysteine residues (Obata et al., 2011). Furthermore, both FUM1 and FUM2 isoforms can be oxidized when exposed to diamide, resulting in a reduction of their enzymatic activity. Notably, the application of dithiothreitol (DTT) has been shown to restore the activity of these enzymes (Zubimendi et al., 2018). Oxidation of FUM results in the formation of high molecular mass aggregates lacking enzymatic activity. Interestingly, the oxidized form of FUM1 can be effectively reduced by incubation with leaf extracts and NADPH together, but not by either component alone (Zubimendi et al., 2018). Under mild oxidative conditions, the formation of intermolecular disulfide bridges, such as Cys339‐Cys339, appears to restrict FUM flexibility. This structural change affects the catalytic capabilities of two adjacent active sites (Zubimendi et al., 2018), highlighting the complex interplay between redox status and FUM activity.

Interestingly, FUM activity has been found to accumulate to higher levels in mitochondrial extracts from *trxo1* and *ntra ntrb* backgrounds compared to wild‐type Arabidopsis plants. The addition of recombinant Trx*o1* to the mitochondrial extracts markedly suppressed FUM activity across all genotypes, whereas Trx*h2* addition substantially enhanced it (Daloso et al., 2015). Yet, the stimulating effect of Trx*h2* is unlikely to apply *in vivo* as this isoform does not localize to the mitochondrial matrix in Arabidopsis (Hou et al., 2021; Meyer et al., 2021). The regulation of FUM by the o‐type Trxs of the mitochondrial matrix may represent a key control point in the TCA cycle, allowing for effective throttling of carbon flux to balance resource investment with needs for carbon skeletons and usable energy.

### Mitochondrial malate dehydrogenase

Mitochondrial malate dehydrogenase (mMDH; EC 1.1.1.37) catalyzes the interconversion of malate and OAA using NAD^+^/NADH as cofactors (Figure 1). The direction of this reaction is influenced by cellular requirements and the NAD redox status in the mitochondrial matrix (Sweetlove et al., 2010). Additionally, mMDH participates in shuttle systems that facilitate the efficient transport of OAA and malate between different cellular compartments (Dao et al., 2022).

In Arabidopsis, two mMDH isoforms exist, with mMDH1 being the predominant form (Tomaz et al., 2010). mMDH activity was observed to be controlled in a thiol redox‐independent manner and rather by adenine nucleotides *in vitro* (Yoshida & Hisabori, 2016), even though an active Cys‐redox switch was identified in a mitochondrial Cys‐redox‐switch‐ome (Nietzel et al., 2020). Recent studies have revealed that lysine acetylation plays a significant role in regulating mMDH activity across various plant species. Arabidopsis mMDH1 has been found to be *acetylated* at four lysine residues (170, 325, 329, and 334) (König et al., 2014), while *Physcomitrium patens* (*P. patens*) mMDH1 has only one acetylation site (Lys172) (Balparda et al., 2022). The conservation of acetylation at Lys170 in Arabidopsis mMDH1 and Lys172 in *P. patens* mMDH (corresponding to Lys169 in Arabidopsis) across many plant species suggests that lysine acetylation is a common mechanism for fine‐tuning mMDH activity (Balparda et al., 2022). The effects of lysine acetylation on mMDH activity vary depending on the specific residue and plant species. In the direction of OAA reduction, acetylation of Arabidopsis mMDH1 at Lys169, Lys170, and Lys334 reduces enzyme efficiency. Acetylation at Lys169 significantly decreases OAA affinity (higher *K*_m_), while acetylation at Lys170 increases OAA affinity (lower *K*_m_) but reduces turnover number. Acetylation at Lys334 decreases the turnover number. In contrast, acetylation of *P. patens* mMDH1 at Lys172 increases activity by doubling the turnover number. For malate oxidation, acetylation of Arabidopsis mMDH1 at Lys334 strongly activates the enzyme, while acetylation of *P. patens* mMDH1 at Lys172 has no effect on its activity. The diverse effects of acetylation on enzyme kinetics and substrate affinity demonstrate its importance in fine‐tuning mMDH activity under different conditions and in various plant species (Balparda et al., 2022).

### NAD‐malic enzyme

NAD‐dependent malic enzyme (NAD‐ME; EC 1.1.1.39) manages mitochondrial malate metabolism jointly with MDH. NAD‐ME catalyzes the oxidative decarboxylation of malate to pyruvate and CO_2_, resulting in the production of NADH (Figure 1). In plants, NAD‐ME predominantly functions as a heteromer consisting of α (NAD‐ME1) and β (NAD‐ME2) subunits, which show high sequence identity (Hüdig et al., 2022; Tronconi et al., 2008, 2020).

In a mitochondrial redox‐proteomic study, four cysteine residues of NAD‐ME2 were identified as being *redox switched*, indicating their potential role in the enzyme's regulation under varying redox conditions. Activity assays of NAD‐ME in Arabidopsis extracts using DTT‐dithian‐buffers adjusted to different redox potentials suggested that NAD‐ME activity increases under reducing conditions (Nietzel et al., 2020).

## CONCLUDING REMARKS

PTMs play a pivotal role in regulating central carbon metabolism in plants, as evidenced by the examples discussed in this review. Several key enzymes and metabolic processes illustrate unique aspects of PTM‐mediated regulation. For instance, chloroplastic GAPDH exemplifies how PTMs can modulate primary metabolic functions, enable moonlighting roles beyond core metabolism, and integrate diverse cellular signals to fine‐tune enzymatic activity. The PDC, with PTM‐based regulation at the nexus between cytosolic and mitochondrial carbon metabolism, functions as a key metabolic gateway, effectively linking cytosolic glycolytic flux to mitochondrial energy transformation. Other enzymes such as ENO, ACO, and SDH showcase the importance of redox‐based PTM regulation, though further research is needed to fully elucidate these mechanisms.

The regulation of Trx activity presents an intriguing contrast between chloroplasts and mitochondria. In chloroplast stroma, Trx activity is readily linked to photosynthetic activity via redox potential. However, in the mitochondrial matrix of vegetative tissues, the regulation of Trx proteins remains less clear, as there is currently no reliable evidence for changes in matrix NADPH supply and NADPH/NADP^+^ status under normal growth conditions. In principle, such changes could be triggered by decreased electron influx, for example, from metabolism, or increased electron efflux, for example, due to antioxidant defense, even though recent *in vivo* measurements in the cytosol suggest that the NADP redox status is remarkably robust (Scherschel et al., 2024). Changes in NADP status have the capacity to limit reductant supply of the Trx system via NADP‐dependent thioredoxin reductase. Interestingly, such a condition was observed during imbibition of orthodox seeds, where the onset of metabolic flux through NADP‐dependent dehydrogenases of the mitochondrial matrix was found to initiate a feed‐forward cascade of Trx‐mediated redox regulation of TCA cycle flux through sudden Trx reduction (Nietzel et al., 2020).

The study of PTMs in glycolysis and the TCA cycle is a rapidly evolving field of research. Despite the progress in proteomic technologies, which are continually revealing novel PTM targets and modification sites, significant knowledge gaps persist, particularly in areas such as plastidial glycolysis. Comprehensive analysis is required to fully map the PTM landscape and understand its implications for plant central carbon metabolism and beyond. To advance our understanding, several areas deserve specific attention. First, careful assessment of the occurrence of a PTM at a specific site is essential. This includes the identification and cataloging of PTMs across all glycolytic and TCA cycle enzymes, as well as investigation of tissue‐specific and developmental stage‐specific PTM patterns. Second, mechanistic analysis is crucial to elucidate the stimuli that trigger specific PTMs and determine the metabolic consequences of individual modifications as well as their specific combinations. Finally, system‐level analysis will be vital to develop models predicting how PTM‐mediated changes cascade through metabolic networks.

The potential for translational research in this field is significant. Applying insights from PTM studies to develop climate‐resilient crops and exploring the transfer of beneficial PTM strategies from resilient plant species to major crops could have far‐reaching implications for agriculture and food security. Moreover, PTM‐based strategies represent a largely untapped resource for regulating plant metabolism and biosynthesis, opening new avenues in metabolic engineering and synthetic biology approaches.

## CONFLICT OF INTEREST

The authors declare no conflicts of interest.

## Data Availability

Data sharing not applicable to this article as no datasets were generated or analysed during the current study.
